# Broadening the Boundaries of Integrated Care in Response to Necessity: Where Are the Limits for Each Sector, and Who Should Pay for What? Comment on "The Effect of Integrated Care After Discharge From Hospitals on Outcomes Among Korean Older Adults"

**DOI:** 10.34172/ijhpm.9084

**Published:** 2025-05-03

**Authors:** Jonathan Stokes

**Affiliations:** ^1^MRC/CSO Social and Public Health Sciences Unit, School of Health and Wellbeing, College of Medical, Veterinary and Life Sciences, University of Glasgow, Glasgow, UK.; ^2^Danish Centre for Health Economics, Department of Public Health, University of Southern Denmark, Odense, Denmark.

**Keywords:** Integrated Care, Healthcare Expenditure, Health Systems, South Korea

## Abstract

Impacts of integrated care interventions, particularly on utilisation and financial outcomes, can be mixed, sometimes quite disappointing when compared to expectations. Positive deviants come along occasionally, but it is extremely difficult to unpick exactly why one intervention might "work" where others have not. Choi and Yoo evaluated a programme in Korea, which appears to have increased time older patients discharged from hospital spend at home, reduced their odds of a subsequent emergency admission, and decreased total expenditure, although re-admissions increased. The programme stands out particularly in its breadth of non-traditional care activity, home-based primary care and long-term (social) care services, but also broader activities such as nutrition support (eg, meal delivery), movement assistance, lifestyle education, housekeeping, and even home repair. In this commentary, I discuss this broadening of interventions to capture more social determinants of health, ask where boundaries of each sector/service should lie, and who should pay for what.

 The integrated care literature, while theoretically promising, can often make for rather disheartening reading when it comes to empirical results.^1,2^ We all know health systems around the world are struggling with increasingly older populations with more care needs, and the resource implications this has, particularly acute when national debt is spiralling, and fiscal budgets are tightening. The problem is clear, the solution(s) much less so. It is uplifting, then, to find examples of integrated care evaluations that appears to suggest some more positive results.

 In this issue, Choi and Yoo report on a Korean pilot integrated care programme aimed at older adults who have been discharged from hospital, evaluated over a 3-year follow-up (2019-2022).^3^ The programme was a multi-pronged intervention, involving cooperation between medical institutions and the local government. Intervention activities included home-based primary care and long-term (social) care services, but also broader activities such as nutrition support (eg, meal delivery), movement assistance, lifestyle education, housekeeping, and even home repair. The authors exploited the gradual roll-out of the intervention (initially to 13 of 229 local government areas) combined with propensity score matching and a difference-in-difference approach (and instead a cox-proportional hazard model in post-period only to evaluate readmissions). They found that the treatment effect on the treated (after excluding those who did not utilise the integrated care services despite being registered) was, on average:

1. 35.2 (95% CI: 30.7, 39.8) additional days at home per person over the post-period; an 11.6% increase compared to baseline, where the control group went slightly in the opposite direction instead.
2. US$ 6960 (95% CI: -7924, -5996) reduced average total annual expenditure per patient (patient co-payments + insurer’s payments); a massive 40.6% decrease compared to baseline, again control group went slightly in the opposite direction.
3. 44% lower odds (odds ratio of 0.56; 95% CI: 0.48, 0.65) of an emergency visit in the post-period, where the control group went in the same direction but not as much of a reduction.
4. But, a hazard 3.53 times as high for readmissions for the same diseases within 1-year (95% CI: 2.98, 4.19).

In summary, they found that even if patients began coming back to hospital more frequently, they were spending more of their total time at home (as opposed to hospital or care homes), and not arriving as frequently as an emergency case, altogether resulting in total expenditure reductions.

 Before I go into the positives, I do first want to raise some points of caution when interpreting these findings, particularly with such large and positive effect sizes. Firstly, while the methods employed are relatively strong, the matching to a control group did not include pre-intervention outcomes which might have captured some of the unobservable (/unmeasured) factors, and the authors do highlight in the limitations section that there remained slight differences between the groups being compared. While difference-in-difference should wash some of this concern out, requiring parallel trends rather than identical levels, there is no presentation of the pre-trend differences in the article: the critical assumption for this method to be valid. There may well be some bias left in there then, and it’s difficult to tell which direction that might lean. Furthermore, even if fully accepted as given, an average treatment effect on the treated is likely to be larger than the actual impact on the population (average treatment effect) in this case. The realities of people who will not engage (a group excluded from this analysis), for instance, would likely water down any impact when rolled out more broadly.

 Nevertheless, there are good reasons to think some of the elements of the intervention implemented here might be more effective than other integrated care examples reported in the literature. People naturally do tend to need more care as they age. A neglect of this fact has sometimes been the “failure” of previous integrated care interventions (or, at least, naivety in terms of the policy expectations). For example, despite commonly aiming for reduced total expenditure, these programmes can sometimes increase expenditure (at least in the short-/medium-term), which might be caused by an increasing awareness and addressing of previously unmet care needs.^4^ Frequently, these unmet needs of complex (high-cost) patients are described as extending beyond the medical domain, to factors such as isolation, poor housing or living arrangements, and other socio-economic issues. Or, linked to these social issues potentially, to mental health needs which might be particularly challenging to address and face a lack of medical treatment options.^5^

 The inclusion of multiple traditionally non-healthcare activities in the intervention evaluated by Choi and Yoo particularly struck me.^3^ Housekeeping, home repair, food delivery and similar activities go beyond where many integrated care interventions do. While directly addressing some of the housing, nutrition, or other social determinants of health that might drive a lot of the health and long-term care utilisation,^6^ there are also potential indirect effects of these extra activities. Depending on how they are delivered, for instance, potentially addressing some of the isolation and loneliness issues, which might otherwise drive some types of healthcare seeking behaviour as a coping mechanism.^7^ Additionally, the ongoing home-based primary care service contacts may well have been able to identify issues early – avoiding emergency visits perhaps but still directing to appropriate care in a more planned way (eg, increasing elective re-admissions).

 The setting of South Korea is of course notable in this respect of integrating relatively broad care activities. It has recently been labelled a “super-aged” society, with roughly 20% of the 51 million people in the country now over the age of 65. The country’s fertility rate gets regular attention, well below replacement, begging questions of who will provide the care and support to this increasingly aging population.^8^ It is perhaps no surprise, then, that “necessity is the mother of invention.”

 As usual with these multi-pronged, complex interventions, though, trying to work out the magic ingredient(s), which components actually did what, is ultimately a black box, as the authors acknowledge. They did speculate, though, that the decreased total expenditure was likely due to the increased home stays outcome (so, inversely, indicating decreased stays in hospitals and long-term care facilities). This seems likely, although the decrease in emergency visits also seems likely to contribute here. When measuring expenditure, the “emergency” tag tends to attract a higher price in many healthcare systems, even for equivalent healthcare resource groups (/diagnosis-related groups).^9^ That price difference might (and likely mostly does) reflect actual higher *costs *(the actual resource consumption, eg, staff time, equipment, medications), but not necessarily for every occasion and precisely capturing the actual difference. Simply re-labelling/pricing the same care from emergency admissions to elective could result in expenditure changes (even if not necessarily as large underlying cost changes). For example, if home-based primary care was able to catch an issue and refer even a day prior to when the same case would otherwise have entered via the emergency room.

 The other important point to consider when interpreting the striking total expenditure results reported here is the boundaries of the different providers and payers involved. On the payer side, the total expenditure measure used here can potentially be a bit problematic. This measure includes both insurer payments, but also patient co-payments. Patients will definitely have a preference for whether it is their own pockets which will be affected by any potential savings, or the insurer’s! Similarly, societally, it is not only the healthcare budgets alone that matter. While decreasing expenditure in parts of healthcare, there is presumably increased expenditure elsewhere – all the extra integrated care services provided by the local government could not be costed or included in the “total” expenditure in this study, for instance. Again, this broader perspective should increasingly matter for providing integrated care services – each service will have its own existing providers/payment models/co-pays for patients. If these are changing as part of the integrated care intervention – for example, the healthcare sector is now going to provide additional new social activities which used to be provided elsewhere or privately sought by patients – then there are also potential workload implications for usually over-stretched services. Furthermore, there will likely be political implication, what the general public are willing to have their taxes pay for, for instance.

 As we increasingly acknowledge the wider system needed to keep us healthy, we also need to work out the (new) boundaries of each sector, how they all fit together to deliver patient and system outcomes, and who pays what/when. This is a major challenge for even those interventions which appear to show initial promise as they scale and spread to new contexts.

## Ethical issues

 Not applicable.

## Conflicts of interest

 Author declares that he has no conflicts of interest.
